# Implementing monitoring technologies in care homes for people with dementia: A qualitative exploration using Normalization Process Theory

**DOI:** 10.1016/j.ijnurstu.2017.04.008

**Published:** 2017-07

**Authors:** Alex Hall, Christine Brown Wilson, Emma Stanmore, Chris Todd

**Affiliations:** a School of Health Sciences, Faculty of Biology, Medicine and Health, University of Manchester, and Manchester Academic Health Science Centre, Oxford Road, Manchester, M13 9PL, UK; b School of Nursing, Midwifery & Social Work, University of Queensland, Brisbane, Australia; c Central Manchester University Hospitals NHS Foundation Trust, and Manchester Academic Health Science Centre, UK; d University Hospital of South Manchester NHS Foundation Trust, and Manchester Academic Health Science Centre, UK

**Keywords:** Ambulatory monitoring, Assistive technology, Case study, Dementia, Implementation, Normalization process theory, Long-term care, Residential facilities, Qualitative research, Uptake

## Abstract

**Background:**

Ageing societies and a rising prevalence of dementia are associated with increasing demand for care home places. Monitoring technologies (e.g. bed-monitoring systems; wearable location-tracking devices) are appealing to care homes as they may enhance safety, increase resident freedom, and reduce staff burden. However, there are ethical concerns about the use of such technologies, and it is unclear how they might be implemented to deliver their full range of potential benefits.

**Objective:**

This study explored facilitators and barriers to the implementation of monitoring technologies in care homes.

**Design:**

Embedded multiple-case study with qualitative methods.

**Setting:**

Three dementia-specialist care homes in North-West England.

**Participants:**

Purposive sample of 24 staff (including registered nurses, clinical specialists, senior managers and care workers), 9 relatives and 9 residents.

**Methods:**

36 semi-structured interviews with staff, relatives and residents; 175 h of observation; resident care record review. Data collection informed by Normalization Process Theory, which seeks to account for how novel interventions become routine practice. Data analysed using Framework Analysis.

**Results:**

Findings are presented under three main themes: 1. Reasons for using technologies: The primary reason for using monitoring technologies was to enhance safety. This often seemed to override consideration of other potential benefits (e.g. increased resident freedom) or ethical concerns (e.g. resident privacy); 2. Ways in which technologies were implemented: Some staff, relatives and residents were not involved in discussions and decision-making, which seemed to limit understandings of the potential benefits and challenges from the technologies. Involvement of residents appeared particularly challenging. Staff highlighted the importance of training, but staff training appeared mainly informal which did not seem sufficient to ensure that staff fully understood the technologies; 3. Use of technologies in practice: Technologies generated frequent alarms that placed a burden upon staff, but staff were able to use their contextual knowledge to help to counter some of this burden. Some technologies offered a range of data-gathering capabilities, but were not always perceived as useful complements to practice.

**Conclusion:**

Implementation of monitoring technologies may be facilitated by the extent to which the technologies are perceived to enhance safety. Implementation may be further facilitated through greater involvement of all stakeholders in discussions and decision-making in order to deepen understandings about the range of potential benefits and challenges from the use of monitoring technologies. Staff training might need to move beyond functional instruction to include deeper exploration of anticipated benefits and the underlying rationale for using monitoring technologies.

**What is already known about this topic?**

• Monitoring technologies may be appealing to the care home sector to help enhance safety, increase resident freedom, reduce staff burden, and reduce costs, although robust evidence for their clinical and cost effectiveness is lacking.
• There may be a range of challenges to the implementation of such technologies, including removal of wearable devices by residents, generation of false alarms, and false senses of security in technologies that lack reliability.
• There are ethical concerns about the use of such technologies, including their influence upon residents’ freedom, autonomy, human rights, privacy, and dignity, the potential dehumanising of person-centred care, and the potential for remote monitoring by management of staff activity.

**What this paper adds**

• The overwhelming justification for the use of monitoring technologies is likely to be made based on the extent to which they are perceived to enhance safety, with less consideration about other potential benefits or challenges.
• The involvement of stakeholders in discussions and decisions around monitoring technologies seems to be variable: staff training tends to be informal and based upon assumptions that technologies will be simple to use, and the involvement of residents is particularly challenging due to the impacts of cognitive impairment.
• Greater involvement of stakeholders in discussions and decisions, and staff training that goes beyond functional instruction, may help to facilitate deeper understanding of benefits and challenges from using monitoring technologies in practice.

## 1 Background

Today’s ageing populations are associated with increasingly large numbers of people with dementia and complex co-morbidities, who are progressively reliant upon residential care facilities for long-term support (OECD/European Commission, 2013, Office for National Statistics, 2014). In the UK (and the present paper), the term ‘care home’ refers to facilities providing 24-h residential care, which may include nursing care (British Geriatric Society, 2011). Recent decades have seen improvements in quality of care in many care homes (Owen et al., 2012). However, the sector is facing extremely complex challenges, including limited resources, problems with workforce recruitment and morale, poor public image (Lievesley et al., 2011, Alzheimer’s Society, 2013), wide variations in quality (Care Quality Commission, 2016), and diverse and unclear models of health service delivery (Goodman et al., 2016). In recent years, UK health and social care policy has recognised the need for innovation within the care home sector (Commission on Residential Care, 2014); better integration of care homes into the wider healthcare system (NHS England, 2016); and higher care standards, staff knowledge and skills (Department of Health, 2009, Department of Health, 2015). Global policy emphasises the potential of technological innovation to enhance clinical outcomes, economic benefits, and patient experience (Howitt et al., 2012, World Health Organization, 2016). Such innovation may be particularly appealing for the care home sector given the challenges it faces (Westphal et al., 2010). Table 1 shows a range of available technologies which may enhance quality of care in care homes.

**Table 1:** Examples of technologies which may enhance quality of care in care homes for people with dementia (adapted from Alzheimer’s Society, 2015, Bharucha et al., 2009).

Technology	Description	Examples
Cognitive aids		
Prospective memory aids	Artificial intelligence devices delivering reminders or procedural guidance as necessary to wearer for task completion	Reminder messages; clocks and calendars; automatic pill dispensers

Retrospective memory aids	Devices to show historical events to stimulate autobiographical memory	Multimedia software to show films and photographs of historical events; camera which passively takes photographs whilst worn by person with dementia


Physiological sensors		
Vital signs and metabolic parameters	Measurement of parameters, with potential to alert relatives or care staff to signs of adverse medical conditions	Bed sensors to measure heart rate or detect seizures

Fall detectors	Detection of falls, either manual (requires faller to activate alert after fall) or automatic (fall event triggers alert)	Body-worn sensors e.g. accelerometer on hip

Environmental sensors	Low-cost sensors to measure single or multiple factors	Acoustic; pressure; motion; e.g. may switch on lights automatically

Advanced integrated sensor systems	Combined system to detect and provide alert to adverse event (e.g. fall).	Usually comprised of control panel, various environmental sensors and alert device for caregiver (e.g. alarm or pager alert)

Wearable radiofrequency transmitters	Radio frequency identification [RFID] system to monitor location, movement and activity	Usually comprised of tag worn by person with dementia, and sensors installed within building

Satellite-enabled technology	Tracking devices able to trace a missing person in order to promote safer walking	GPS-enabled smartphone

Video-based systems	Video cameras to stream or record activity and behaviour	CCTV

All of the sensors, integrated systems, radio frequency, satellite and video-based systems in Table 1 may be categorised as ‘monitoring’ technologies (Cahill et al., 2007, Niemeijer et al., 2010). These technologies may potentially increase safety, enhance clinical knowledge, reduce staff burden, and promote freedom of movement for residents; outcomes which may be particularly desirable in long-term dementia care (e.g. Rantz et al., 2013, Woolrych et al., 2013). However, robust high quality evidence demonstrating the effectiveness of monitoring technologies in achieving such outcomes is lacking (Khosravi and Ghapanchi, 2015, Schoenfeld et al., 2016), and there is a lack of evidence to support claims that they will be cost-effective for dementia care (Gibson et al., 2016). Robust data regarding usage trends and distribution of monitoring technologies throughout the UK are lacking, however the most common type in use and desired by care homes seems to be fall detectors (South East Health Technologies Alliance, 2016).

Despite limited evidence for the effectiveness of monitoring technologies, dementia policy tends to emphasise their potential benefits uncritically, and implementation is strongly encouraged (Gibson et al., 2016). Uptake of technologies into routine healthcare practice is frequently acknowledged as a major challenge, for reasons including cost, time, resistance to change, and user acceptance (Eccles et al., 2009, National Institute for Health and Care Excellence, 2015). A detailed understanding of implementation challenges is important because they may underpin any apparent lack of clinical effectiveness (Medical Research Council, 2008). Monitoring technologies present a range of implementation challenges, such as removal of wearable devices by residents, generation of false alarms and overburden for staff from ‘alarm fatigue’, or creation of a false sense of security (Niemeijer et al., 2010). There are also ethical concerns, including the potential for negative influence on residents’ freedom, autonomy and privacy; for dehumanising care; and for remote monitoring of staff by management (Robinson et al., 2007, Niemeijer et al., 2010). Attitudes towards monitoring technologies are culturally sensitive, for example, there is more scepticism and debate in Europe than North America (Niemeijer et al., 2010).

Research exploring the implementation of monitoring technologies in care homes has largely investigated hypothetical scenarios, such as perspectives on potential use (Robinson et al., 2007, Niemeijer et al., 2010). More recently, literature within health and social sciences and engineering and computer sciences reports upon projects involving real-world implementation of monitoring technologies in care homes (e.g. Zwijsen et al., 2012, Sugihara et al., 2015, Niemeijer et al., 2014, Niemeijer et al., 2015). This literature shows that there seems to be more emphasis placed upon safety, which may be easier to ‘see’ than other potential benefits such as freedom of movement in residents with dementia and concomitant physical impairments. Ethical acceptance of technologies by staff may come from relativist positions such as a lack of objection or awareness from residents, the intention behind the use, or priorities of staff roles. However, the literature lacks detailed insight into processes such as staff training, communication, decision-making and consent around the use of monitoring technologies. It is also largely uninformed by implementation science theory, use of which has been recommended to help develop understandings of the mechanisms underpinning implementation success or failure, and of the contexts in which implementation occurs (Greenhalgh et al., 2004).

The increasing availability, affordability and sophistication of monitoring technologies, and continual encouragement of their use, coupled with a lack of knowledge about context-specific implementation challenges, presents a pressing need for comprehensive exploration into factors influencing the implementation of such technologies within care homes. This paper presents findings from a qualitative study that explored facilitators and barriers to the uptake of monitoring technologies into routine practice in care homes. In particular, we wanted to explore the influence of the ethical debate between ‘safety’ and ‘freedom’, the perception of benefits from using monitoring technologies balanced against the potential challenges such as false alarms, and organisational processes such as training, communication and decision-making. We used Normalization Process Theory (May et al., 2015; see Methods) to add theoretical depth.

## 2 Methods

### 2.1 Design

We used an embedded multiple-case study design (Yin, 2009) within three care homes specifically for people with dementia in North-West England. The case was defined as the process of implementation of monitoring technologies, occurring within the context of a particular care home. The embedded units of analysis were:

• the perspectives of staff, residents and relatives, because relationships among these three groups are integral to care home life (Brown Wilson et al., 2009)
• organisational documentation (e.g. care records), to gain a fuller picture of the relationship between the implementation case and its care home context
• technology manufacturer literature (e.g. product websites; training materials), to consider how monitoring technologies might be promoted to care homes.

### 2.2 Theoretical perspective

We applied Normalization Process Theory (May et al., 2015), which focuses upon the interactions between individuals and organisational contexts to explore the implementation of novel interventions as part of routine components of everyday practice. Normalization Process Theory comprises four generative mechanisms: (i) **Coherence** focuses upon participants’ understanding of the intervention prior to working with it; (ii) **Cognitive Participation** focuses upon the extent to which participants are committed to working with the intervention; (iii) **Collective Action** focuses upon the efforts involved in using the intervention in practice; (iv) **Reflexive Monitoring** focuses upon participants’ evaluations and appraisals of the intervention. Table 2 provides further illustration of these mechanisms.

**Table 2:** Mechanisms of Normalization Process Theory (adapted from May et al. 2015).

Coherence: understanding	Cognitive Participation: involvement
• Is it different from our other interventions? • Do we agree on the anticipated benefits? • Is it compatible with our broader values, ethics and priorities? • Do we understand what we have to do to use it?	• Are there key people influencing it? • Do we feel we can and should contribute? • Can we organise ourselves to contribute? • Can we define how we will use it?
Collective Action: ‘doing’ in practice	Reflexive Monitoring: appraisal
• How successfully can we work with it? • Do we have the right training and skills? • Does our organisation support its use? • Do we trust the technology?	• Can we see its impact? • Do we think its impact is useful? • How do we evaluate it? (i.e. practice and process of evaluation) • Can we adapt it to suit our needs, or adapt our practice as a result of using it?

Normalization Process Theory emphasises the reciprocal nature between these generative mechanisms, since implementation work is iterative rather than linear. Normalization Process Theory views implementation as continuous rather than as a final outcome; ‘successful’ implementation might thus be considered the point at which the intervention becomes *‘the way we do things here’* (May, 2013, p1). The theory has been used by health services researchers primarily as a heuristic interpretive lens rather than as a rigid conceptual framework, with increasing diversification of application to include fields such as e-health, telehealth, mental health, chronic health, maternity care, and speech and language services (McEvoy et al., 2014).

### 2.3 Settings

We recruited three dementia-specialist care homes in North-West England, herein given the pseudonyms “Sycamore Lane”, “Conifer Gardens” and “Heather Grove” to maintain confidentiality. All homes were located in urban areas and provided care for people living with dementia. Sycamore Lane and Conifer Gardens were purpose-built, 60-bed homes providing residential care with nursing. Heather Grove was a converted Victorian house with 27 beds providing residential care without nursing. We recruited the homes through local research networks and established relationships, guided by purposive sampling, as they differed in type of care provision, size, ownership model, physical environment, and used different technologies at different stages of implementation (Table 3). The main doors of each home were secured electronically, with Sycamore Lane and Conifer Gardens also having a permanently-staffed reception adjacent to these doors. This meant that residents were not free to leave independently. For residents unable to consent to this arrangement, the UK legal process requires a Deprivation of Liberty Safeguard (see Care Quality Commission, 2016).

**Table 3:** Descriptions of care homes, including site information, staffing levels, technologies used, and data collected.

Care home name and site information	Staffing levels (minimum)	Technologies	Key features of technologies	Data collected
Sycamore Lane • Local chain • For-profit • 60 beds • Residential and nursing care • Purpose-built	Day shift: 1 RN, 3 senior care workers, 6 care workers Night shift: 1 RN, 1 senior care worker, 4 care workers	Advanced integrated sensor system: Nurse call with bed sensors	Call buttons; sensors installed underneath mattresses and plugged into units affixed to headboards. Bed sensors activate upon movement; non-movement e.g. seizure; can be set to delay to account for mobility level of resident. Alarms sent to pagers carried by staff. Central computer records data about alarm (de)activation and resident vital signs e.g. heart rate	• Observation: 73 h • Interviews: 10 staff [1 manager, 2 RNs, 6 senior/care workers, 1 facilities manager], 2 relatives, 1 resident • Care plans: 4 residents • Technology manufacturer literature: 2 websites
		Physiological sensor: Activity tracker	Body-worn sensor clipped to clothing or carried in pocket; continuous monitoring of user activity via accelerometer	

Conifer Gardens • National chain • Non-profit • 60 beds • Residential and nursing care • Purpose-built	Day shift: 2 RNs, 3 senior care workers, 6 care workers, 1 OT, 1 clinical lead Night shift: 1 RN, 2 senior care workers, 4 care workers	Advanced integrated sensor system: Nurse call with pressure mats	Call buttons; pressure mats placed on floors and plugged into units on bedroom walls. Pressure mats activate upon touch. Alarms sent to units fixed on walls in communal areas. Central computer records alarm (de)activation	• Observation: 74 h • Interviews: 11 staff [2 managers, 3 RNs, 2 clinical, 4 senior/care workers], 6 relatives, 1 resident • Care plans: 4 residents • Technology manufacturer literature: 2 websites, 1 training manual
		Radio-frequency identification: Location-based system	Fobs worn by residents; wireless sensors installed in ceilings. Alarms sent to pagers carried by staff. Central computer records data about location and mobility of residents and staff	

Heather Grove • Local chain • For-profit • 27 beds • Residential care only • Converted house	Day shift: 1 senior care worker, 3 care workers Night shift: 2 care workers	Advanced integrated sensor system: Nurse call with pressure mats	Same system as Conifer Gardens	• Observation: 28 h • Interviews: 3 staff [1 manager, 2 senior/care workers], 1 relative, 1 resident • Care plans: 1 resident • Technology manufacturer literature: 2 websites
		Environmental sensors: Door monitors	Environmental sensors affixed to bedroom doors; recording of time and duration of opening of each door via magnetometer; data logged in ‘cloud’ and accessible from laptop in managers’ office	

### 2.4 Participants: sampling and recruitment

We recruited staff members, relatives, and residents according to the following inclusion criteria: any involvement with monitoring technologies in the care home (including refusal); over age 18; able to communicate in English. We recruited participants using a purposive sampling approach. Staff participants had a wide variety of roles, responsibilities, shift patterns, and lengths of employment within the homes. Resident and relative participants had differing lengths of experience of life within the homes, and residents had diverse care needs and levels of cognitive impairment. Within each home, the first author (AH) introduced himself to as many staff as possible, explained the purpose of the study and gained familiarity with the environment. Following initial periods of acclimatisation, potential participants were identified and invited for interview. For example, where there were certain residents whose care involved use of particular technology, staff with key roles within these residents’ care were identified. We identified further participants in an iterative process according to emerging findings and reflections.

### 2.5 Data collection

Non-participant observations were conducted to explore routine practice (Fitzpatrick and Boulton, 1994). Observations occurred primarily in communal areas, and included daily routines such as morning and evening bedtimes, shift handovers, medication rounds, and meal times. Some observations occurred within bedrooms of resident participants in order to see components of monitoring technologies; these only occurred when the resident granted permission, were usually accompanied by a relative, and did not include delivery of personal care. Each observation period typically lasted three to four hours. Brief notes were taken during observation periods, and typed up into fuller field notes within 24 h. Observations were conducted within all shift patterns over the 24-h period of daily care.

Semi-structured interviews were conducted with staff and relatives, guided by the mechanisms of Normalization Process Theory as a heuristic device. Interview prompts are provided as Supplementary material. Participants were also asked to comment on observational data (Silverman, 2005, Brown Wilson and Clissett, 2010). Participants were interviewed individually, except for two joint interviews each with two participants. Interviews were arranged around staff availability during working hours, and relatives’ visitation patterns. Most interviews took place in quiet spaces, recorded on an encrypted digital recorder, although a minority were conducted in more open areas and recorded using handwritten notes. Audio-recorded interviews were transcribed verbatim, and handwritten interview and conversation notes were typed up, within three to four days. Interviews with staff members lasted from 22 to 90 min, most often around 40 min; with relatives from 16 to 35 min, most often around 25 min. Informal conversation with residents with dementia is a complementary method to more formal interview, since it may be tailored to cognitive ability and guards against privileging those able to participate in long conversation (Bamford and Bruce, 2000). Informal conversations with residents were held to the extent to which their levels of cognitive ability would allow, and recorded via handwritten notes.

Care records of resident participants were consulted, with a standardised form used to extract information about technologies used, the implementation decision (use or withdraw), the date of the decision, who made the decision, and the reason for the decision. Agreement was sought from management, and consent from residents and their consultees (see Consent and ethics approval), to access these data. Other documentation consulted included a training manual for one of the technologies at Conifer Gardens, and manufacturer websites relating to the technologies within each home. Notes were taken on relevant detail from the training manual and manufacturer websites.

All data were collected by the first author (AH). He has prior experience working and researching in social care settings, but not within dementia care, and is not a clinician. AH regularly discussed emerging findings and experiences with the other authors, two of whom (CBW and ES) are Registered Nurses with experience of working and researching in residential dementia care. Data collection continued until saturation, determined as the point at which no new findings were generated during the development of the analytic framework (O’Reilly and Parker, 2012).

### 2.6 Data analysis

**Fig. 1:**
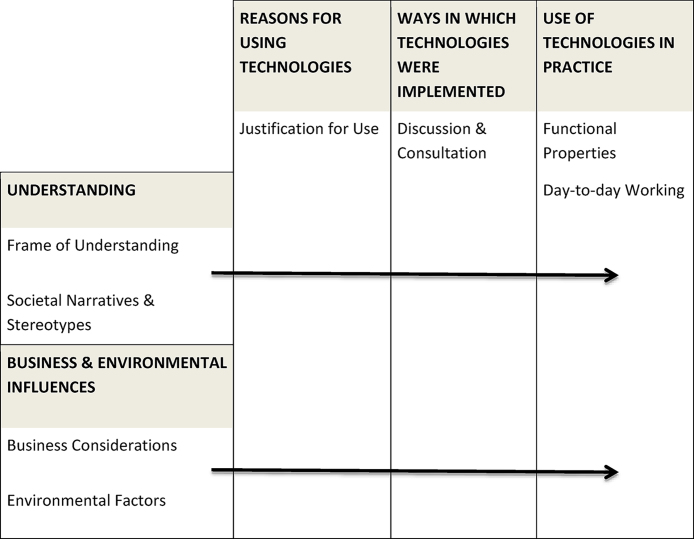
The five inter-related themes and eight sub-themes arising from framework analysis.

Data were analysed using the Framework Analysis approach, which involves rigorous and transparent charting of coded data via analytical matrices (Ritchie et al., 2003), supported by NVivo 10 software. This approach aligns with the systematic approach advocated by Yin (2009) for multiple-case study designs. Analysis began with familiarisation and coding of a small subset of the data to develop a working analytic framework, which was then applied to the remaining data and refined in an iterative process. The development of codes and themes involved a combined inductive and deductive approach. The interview topic guide was informed by Normalization Process Theory, and hence its mechanisms were present within the data, but they were not used to pre-select codes and themes. Coding was conducted in a manner which allowed exploration of issues highlighted by Normalization Process Theory (i.e. participants’ understandings, involvement, use, and evaluations of the technologies) and by previous literature (i.e. emphasis upon safety; ethical questions; staff burden; false alarms and fatigue). However, the analysis left enough room and openness to explore unexpected elements within the data (Gale et al., 2013). These stages generated a final analytic framework of 49 codes, grouped and charted into eight sub-themes. The final stage of analysis involved interpretation of these eight sub-themes, during which further relationships between the sub-themes became clearer, resulting in the final organisation of the eight sub-themes into a matrix consisting of five inter-related themes (Fig. 1).

### 2.7 Quality assessment

We used multiple methods of data collection from different sources (e.g. interviews with key individual participants, observations of daily practice, and data extracted from care records) which were triangulated to provide analytic depth, comprehensiveness and reflexivity (Mays and Pope, 2000). The analytic process was systematic, transparent and reflective: the first author led the coding of the data and regularly checked the analysis with the other three authors as the analytic framework was developed; the first author developed further interpretation of the results with regular critical comment from the other three authors. In this paper we provide clear descriptions of the sampling framework and of the research contexts so that the findings may be transferrable to other settings, and we provide data from a wide range of participants’ voices. All of these factors support high-quality qualitative research (Mays and Pope, 2000).

## 3 Consent and ethics approval

All staff and relative participants were provided with written information and given the opportunity to discuss the study before written consent was obtained. As the homes were dementia-specialist homes, the vast majority of residents lacked capacity to provide written consent, determined principally by staff report. For these residents, the process model of consent was applied (Dewing, 2007). For each resident, this included identifying a consultee (usually a relative) able to advise in principle whether the resident might be interested in taking part. More information was then gathered about that resident’s preferred means of signalling consent (e.g. personal behaviours associated with wellbeing), and the presence or absence of such signals was monitored during every interaction with that resident. Pseudonyms were given to all participants to maintain confidentiality. Ethics approval was granted by the NHS National Research Ethics Service Committee North West – Haydock (reference 13/NW/0752).

## 4 Results

Data were collected between February and November 2014. Data included: 175 h of observation; 36 formal interviews (24 staff, including senior management, registered nurses, and care workers from day and night teams; 9 relatives; 3 residents); data extraction from nine care records, one training manual and five technology manufacturer websites. Two additional relatives declined their own participation and any approach to their family member, as they felt that participation would be too burdensome. Of the nine residents recruited, only three possessed enough cognitive capacity to engage in conversation about technologies in their care. Sycamore Lane and Conifer Gardens were relatively new homes, and the majority of staff had worked in the homes for around two years. Heather Grove had functioned as a care home for longer, under previous ownership, and the majority of staff had worked there for between three and five years. The mean age of staff was 39.75 years (range 21–64; SD 12.62). Most relatives and residents had been involved with the homes for between one and two years. The mean age of relatives was 55.67 years (range 41–78; SD 12.67), and of residents was 81.33 years (range 72–95; SD 8.22). The vast majority of staff, residents and relatives were of White British ethic origin, and female.

Each home was using a nurse-call system which included bed-monitoring capability via under-mattress bed sensors (Sycamore Lane) or on-floor pressure mats (Conifer Gardens and Heather Grove). Sycamore Lane was trialling a wearable activity tracker with one resident; Heather Grove was using door-monitoring technology to record night-time checks on residents by staff; and prior to the study Conifer Gardens had used a wearable radio-frequency location-based system with selected residents. Table 3 provides further detail about the homes, the participants and the technologies.

Findings are presented in the following three sections corresponding to the column headings of the thematic matrix shown in Fig. 1.

### 4.1 Reasons for using technologies

The most common justification provided by staff and relatives, and stated within care records, for using monitoring technologies was to enhance safety. This enhancement came from either mitigating risk of residents falling, or alerting staff to the movement of residents deemed to pose a risk to themselves or other residents in order to prevent physical altercations between residents. The location-based system at Conifer Gardens had reportedly been met with initial excitement amongst staff about its potential to enhance freedom of movement for residents. Yet staff reflected that in practice it had seemed more useful for preventing physical altercations between residents. Some interviewees, including relatives, cited the potential for bed-monitoring technologies to provide benefits to residents in addition to enhanced safety, such as increased privacy from not needing continuous human observation. Some staff members highlighted the role played by the technologies in directing their attention to where it was most needed at the right time, which was felt to be an indicator of compassionate care. However, the consensus suggested that considerations such as positive or negative impacts upon resident privacy or freedom of movement were slight compared to the need to keep residents physically safe:

*“I’ve got a role in the first place to ensure the safety of the residents… the equipment is vital in keeping them safe, like the [pressure] mat”* (Martha, Nurse (night), Conifer Gardens)

Resident care records repeatedly showed declarations of assurance, stating that pressure mats were implemented “*to ensure”* residents did not fall. Despite this emphasis, most staff recognised that technologies could not guarantee safety, offering comments such as:

*“even though they’ve got the [bed] sensors, people still fall… it’s just to alert you that they’re getting out of bed”* (Doug, Care Worker, Sycamore Lane)

Staff suggested that this understanding seemed less acknowledged among relatives. However, some relatives did understand that bed-monitoring technologies were not guarantees of safety; one described falls prevention as “*the million dollar question”* (Colin, son, Conifer Gardens). There were numerous examples suggesting that relatives favoured use of monitoring technologies on the grounds of enhanced safety even where they may threaten resident privacy.

All three residents interviewed did not appear to be concerned at being monitored, offering comments such as *“when you’re getting older it’s important [for staff] to know where you are”* (George, Sycamore Lane). At Heather Grove, Jack seemed to value privacy from other residents, rather than from staff entering his room when prompted by monitoring technology. He expressed frequent irritation at one resident whom he seemed to find particularly intrusive, saying *“she will be off in a minute, you watch”,* and that he often found her in his room.

The managers at Conifer Gardens had been cautious about the use of monitoring technologies, citing ethical concerns about the potential for a ‘Big Brother’ effect of invasive remote surveillance upon residents. They frequently appraised individual resident need for monitoring technology, and withdrew technology from residents they judged to be no longer in need. They outlined that they had many discussions with staff and relatives about this approach, but that it was not always well understood:

*“I think people see it [pressure mat] as something very, very different… because it’s a safety net… they don’t rely on a safety net of nutritional support, they see that as treatment, but a pressure mat alerts them to when something is wrong… and therefore in their mind’s eye they go ‘well it must stay there’*’’** (Ben, Deputy Manager, Conifer Gardens)

Staff perspectives seemed to be grounded in fears that they would be blamed for accidents or injuries to residents, even though there were no reports of this having happened within the home. The spectre of a blame culture was most apparent at Heather Grove, where the managers had justified implementation of the door-monitoring technology out of fears influenced by media portrayals of care homes.

### 4.2 Ways in which technologies were implemented

At each home, the bed-monitoring technologies were introduced by senior staff to new staff when they commenced employment. These technologies were commonly perceived by management and staff as simple to use, yet some senior staff suggested that a more formal approach to training would be valuable. The location-based system at Conifer Gardens had been introduced to staff in a formal training session run by the manufacturer. Senior staff had received the most training, including how to access data stored on the system’s central computer. Care staff received less training, and were encouraged to use the system as soon as possible on the grounds that *“you cannot do any harm”* (system training manual, Conifer Gardens), but some reported that they would have preferred training that included clarification of the anticipated benefits and rationale for use.

At each home, decisions to implement monitoring technologies with specific residents were ultimately made by senior management. At Conifer Gardens and Heather Grove, staff appeared to be involved in some discussions about use of the bed-monitoring technologies, with decisions about the implementation of pressure mats clearly documented within care records and formal communication mechanisms. At Sycamore Lane there was more of a mixed picture, in which the owner of the home took much more responsibility for implementation decisions, and senior nursing staff appeared to be less involved than at the other homes. Although senior staff suggested that they might discuss provision of bed sensors in shift handovers and team meetings, implementation was not formally documented in residents’ care records and thus it was difficult to see an audit trail. Through a combination of interviews and observations it became apparent that there was a lack of consistent understanding amongst staff at Sycamore Lane about precisely which residents had bed sensors.

At Heather Grove, information about the implementation of the door monitors seemed initially to have been deliberately withheld from night staff so that management could check staff activity. Although all staff had subsequently been informed about the technology, there was a lingering lack of trust, with the deputy manager reporting that she would *“have a quick flick through”* the data before the shift handover from the night staff. The activity tracker at Sycamore Lane was being tested with one resident. Apart from the resident’s key staff team, most staff had not been given a clear introduction to this technology. This appeared to fuel rumours about “*reports that the staff are being lazy”* (Aggie, Senior Care Worker), and that it was going to be used to monitor their activity.

Relatives and residents appeared to be more involved in discussions about technologies at Conifer Gardens than at Sycamore Lane or Heather Grove. Conifer Gardens particularly attempted to respect resident capacity to make a choice, even if that choice was deemed to be unwise. This was exemplified by the approach used with one resident, who had continually refused a pressure mat despite injuring herself falling out of bed:

*“until she loses the ability to make those decisions for herself then we’re not to intervene… people are entitled to make a bad choice”* (Ben, Deputy Manager, Conifer Gardens)

Where residents were capable of engaging in discussion about monitoring technologies, staff reported challenges in ensuring that residents were fully aware of the implications of both the technology and the environment in which they were living:

*“you were trying to explain to [a resident] ‘oh [the location-based system] is so you can have a bit more independence’… at that point [residents] are like ‘well I can go where I want, when I want!”'* (Beatrice, Occupational Therapist, Conifer Gardens)

### 4.3 Use of technologies in practice

#### 4.3.1 Response to alarms

Although interview data generally showed that staff felt the bed-monitoring technologies were perceived as straightforward to use, staff in each home often seemed to forget how to operate the technologies when delivering care to residents. Observations uncovered numerous instances where staff appeared to have inadvertently generated alarms within resident bedrooms. There was also speculation from management that in the moments of delivery of care, staff might inadvertently trigger alarms as their focus was on the resident rather than the technology. The alarms were received by wall units (Conifer Gardens and Heather Grove) or pagers carried by senior staff (Sycamore Lane). In all three homes, alarms were sent to all the wall units or pagers throughout the building, rather than isolated to local areas in which the alert had been generated. Staff seemed to prefer wall units, citing an increased awareness of potential incidents in contrast to a burden placed upon the small number of senior staff responsible for carrying pagers. Staff also appeared to have devised strategies to avoid pagers, including not replacing batteries and claiming that the pagers were broken, or simply ignoring them.

There was therefore a sense of overburden from the frequency of alarms generated within the homes, but the building-wide distribution of alarms was perceived to be preferable to isolated alarms as it raised awareness amongst all the staff of potential incidents. Observational data revealed that staff in all homes frequently did not seem to respond to alarms, but subsequent interviews exploring these observations uncovered that they used local knowledge to make decisions about responses:

*“on our floor we’d know whose alarm could go off, and if they’re not in their room then you wouldn’t bother looking”* (Olivia, Nurse, Conifer Gardens)

This strategy was felt to be a useful heuristic for managing responses to frequent alarms, but the decision to distribute the alarms throughout the buildings was acknowledged by managers to be a difficult one, as it presented the risk of complacency.

The location-based system at Conifer Gardens was reported to have generated a high number of false alarms which seemed to have become an unmanageable burden. Residents may have removed fobs and moved to a different location, which confused staff as the resident was not located where the system indicated. There were also reportedly instances where residents wearing fobs had left the building with relatives, generating alarms which caused staff to panic that they had left the building unattended. At times the home had to employ intricate and burdensome strategies to avoid false alarms:

*“[name of resident] got so used to having that [fob] on that she started to become hyper-anxious when leaving the building because she didn’t have that round her neck… we had to take the batteries out or give her a dud one when she went out the building… [there were] more logistical issues to using it than to just not have it at all”* (Ben, Deputy Manager, Conifer Gardens)

#### 4.3.2 Use of information

The bed-monitoring technologies were felt to be useful in helping staff identify patterns in resident behaviour and explore reasons for these behaviours. The bed sensors at Sycamore Lane were capable of recording clinical data such as heart rate, but the manager reported that *“it’s not something that we use readily”*, and this functionality was never observed in use during the present study. The location-based system at Conifer Gardens was similarly able to record data, including information about resident mobility activity. This functionality had initially been anticipated as potentially useful for enhancing clinical understanding, however, the Occupational Therapist reflected that the time needed to analyse and interpret these data had been *“a job in itself”* and thus has been difficult to integrate into daily practice. There were questions about the clinical utility of some of the data, which appeared to become more pronounced when considering the financial expense of the technology:

*“it was adding another layer of assessment to make a decision when you didn’t necessarily need that data… there’s nothing wrong with traditional observation… we’re not being replaced by a machine… if I wanted to know how many steps people need* [sic] *I can just buy a £4 pedometer”* (Ben, Deputy Manager, Conifer Gardens)

These findings show that patterns of movement or behaviours of residents observed directly by staff in response to technological alarms may have been considered more useful than remote data collected directly by monitoring technologies.

## 5 Discussion

This study involved staff members, relatives and residents at three dementia-specialist residential care homes, to explore how the use of monitoring technologies might become part of routine practice. The primary reason given for implementing monitoring technologies was to enhance safety. This often seemed to override consideration of other potential benefits (e.g. increased resident freedom) or ethical concerns (e.g. resident privacy). Some staff, relatives and residents were not involved in discussions and decision-making processes, which seemed to limit understandings of the potential benefits and challenges from the technologies. Staff training appeared mainly informal, which did not seem sufficient to ensure that staff fully understood the technologies. Technologies generated frequent alarms, but staff were able to use their contextual knowledge to help to counter burden from alarm fatigue. Some technologies offered a range of data-gathering capabilities, but were not always perceived as useful complements to practice, particularly if associated with a high financial cost. These findings are discussed under two broad headings: safety and the ethical debate, and practicalities.

### 5.1 Safety and the ethical debate

The implementation of monitoring technologies seemed to be facilitated primarily by the extent to which they were perceived to enhance safety. This suggested a dominant model of care which accepts the monitoring of residents as a core part of practice, and ascribes less value to developing a shared understanding of additional benefits from the use of technologies, or of ethical issues surrounding their use. This finding is supported by a mixed picture from the literature which highlighted some understanding of a range of simultaneous potential benefits such as increased safety and resident freedom (Engström et al., 2009, Niemeijer et al., 2011, Niemeijer et al., 2015, Sugihara et al., 2015, Zwijsen et al., 2012), but which emphasised a perception that the primary (or only) role for monitoring technologies should be to enhance safety (Aud, 2004, Niemeijer et al., 2014, Zwijsen et al., 2011, Zwijsen et al., 2012). Despite this emphasis, many staff members appeared to recognise that bed-monitoring technologies could not prevent falls, which is supported by other research (Zwijsen et al., 2012). Whilst these technologies may lack effectiveness, they help staff to provide much quicker assistance to residents who have fallen than would otherwise occur from traditional observation rounds, and hence they remain appealing to residential care facilities. The difficulties at Conifer Gardens in helping residents understand freedom of movement from the location-based system develop speculations that such technologies might only improve quality of life for residents in the early stages of dementia with high levels of mobility (Te Boekhorst et al., 2013), as they highlight that there may be challenges in helping such residents understand and accept these technologies.

Some staff justified use of monitoring technologies because of fears of a blame culture within social care. One home used technology for the specific purpose of monitoring staff performance, and there seemed to be a lingering mistrust between staff and management despite staff having been informed about the technology after a brief period of covert use. Staff in other homes were also susceptible to rumours that technologies were being used for this purpose even if not the case. These findings are supported by previous literature (Niemeijer et al., 2010, Robinson et al., 2007, Schikhof et al., 2010, Sugihara et al., 2015) and add to a picture that paints blame as the default response to poor quality health and social care (Baker, 2015). The present study clearly demonstrates that there is a need for greater involvement of staff in discussions about use of monitoring technologies at an early stage of the implementation process to avoid creating a negative culture of an ‘eavesdropping employer’ (Ciocchetti, 2011).

Successful implementation may therefore be enhanced by the involvement of stakeholders within discussions and decisions about implementation in order to allow for deeper understandings of benefits and challenges from using the technologies. From a Normalization Process Theory perspective, this highlights the importance of the relationship between the mechanisms of Cognitive Participation (involvement), Reflexive Monitoring (appraisal) and Coherence (understanding). This relationship was most apparent at Conifer Gardens, where staff, residents and relatives appeared to have reasonably high involvement in the discussion and decision-making process regarding pressure mats (i.e. high Cognitive Participation), underpinned by regular recording and appraisal of use (i.e. comprehensive Reflective Monitoring practice), which contributed to a deeper picture of understanding of benefits and challenges (i.e. a well-developed sense of Coherence). In contrast, at Sycamore Lane, staff, residents and relatives did not seem to make as much contribution to implementation decisions about bed sensors (i.e. there was a lack of Cognitive Participation), and there did not seem to be a clear formal recording of use or appraisal of impact (i.e. there was a lack of clear Reflexive Monitoring practice). This lack of involvement, recording and appraisal appeared to contribute to a scenario in which bed sensors were implemented throughout the home, yet this implementation was undermined by uncertain and varied knowledge about technological distribution, functionality, and relatively little ethical debate or discussion about its alignment with the home’s ethos of care (i.e. there was an uncertain, fragmented Coherence). Niemeijer et al. (2011) identified a perceived importance of clear communication about the purpose and functionality of monitoring technologies. Literature relating to a broader range of interventions within care homes has shown that staff involvement and collaboration in bottom-up implementation processes is a key mechanism for success (Goodman et al., 2016). The use of Normalization Process Theory within the present study shines further light upon the importance of the involvement of stakeholders within successful implementation by showing how it may underpin appraisal work which can develop understandings about the intervention.

### 5.2 Practicalities

Bed-monitoring systems largely contributed to an increase in staff confidence and coordination of practice. Burden from alarm fatigue was primarily a manageable side-effect of the building-wide distribution of alarms. In contrast, the location-based system at Conifer Gardens was beleaguered by false alarms and technical problems, and its full range of data collection capabilities were considered too time-consuming to add value to practice and facilitate successful implementation, particularly when weighed against its financial cost. These findings are supported by the overall picture in the literature which suggests there is a balance between increased confidence, control and coordination in practice delivered by monitoring technologies with overburden from alarms (Engström et al., 2009, Niemeijer et al., 2010, Nijhof et al., 2012, Sugihara et al., 2015). From an ethical perspective, we have discussed above the attractiveness of monitoring technologies for care homes deemed to facilitate fast response to, rather than prevention of, an adverse event. It would seem that this attractiveness depends in part on how far staff are also able to work around alarm burden. Staff generally expressed a desire for more training around monitoring technologies, which concurs with other studies (Engström et al., 2009, Niemeijer et al., 2014).

It is therefore interesting to note that for the bed-monitoring technologies, staff were able to draw upon their knowledge of the local context to overcome the potential barrier to implementation presented by alarm fatigue, and thus seemed to have developed a skilled way of working with these technologies that drew upon their experience rather than formal training. However, some staff had received more formal training around the location-based system at Conifer Garden, but the technical challenges of this system proved to be an insurmountable barrier to successful implementation. From a Normalization Process Theory perspective, these findings show a relationship between the mechanisms of Collective Action (everyday workability) and Reflexive Monitoring (appraisal), whereby the perceived workability of a technology (rather than functional knowledge) leads to appraisals of its worth. For the bed-monitoring technologies, staff received little formal training, but seemed to hold a level of functional knowledge which, enhanced by adaptations to their practice (i.e. adaptive Reflexive Monitoring practice), promoted a workability of the technology sufficient to render it taken up into practice (i.e. sufficient Collective Action). For the location-based system, staff had received more training, yet the majority seemed to perceived the technology as adding little value due to workability challenges caused by technical problems (i.e. Collective Action was hampered by technical issues, which led to negative Reflexive Monitoring).

These findings suggest that simply increasing quantities of functional training may not render a technology any more workable in practice. Rather, training provision may need to include clear discussions about the alignment between the technologies and the values and practices of care within the home so that staff are better able to understand why they are using a technology. From a Normalization Process Theory perspective, this again highlights the importance of the construct of Cognitive Participation (involvement of stakeholders): implementation may be facilitated by greater involvement in discussions to deepen understandings, rather than by simply increasing didactic instruction. This point seems important when emerging technologies of potential value to care homes include advanced, self-learning nurse-call systems (Ongenae et al., 2014), as well as the increased market availability of wearable personal technologies and mobile health apps which are imbued with clinical and ethical questions about safety, effectiveness, and their impact upon the human caring role (Powell et al., 2014, Fox, 2017). The implementation of such technologies may be facilitated by careful and well-planned approaches, in which staff training moves beyond functional instruction to include deeper discussion about the expected benefits and challenges.

### 5.3 Study limitations, challenges, and strengths

There are a number of limitations to our study. First, case study research is not designed to be statistically generalisable (Yin, 2009); second, all three homes were in urban areas; third, the vast majority of participants were females of White British ethic origin. It is possible that facilities situated in more remote areas, or with more diversity, may reveal different views and approaches to the use of monitoring technologies. Nevertheless, findings of an overriding emphasis upon safety, and the higher involvement of stakeholders supporting successful implementation of complex interventions, seem transferrable within the residential care sector (e.g. Brodaty et al., 2014, Chenoweth et al., 2014, Moyle et al., 2013). Fourth, the technologies that were most successfully implemented were the older, more familiar technologies (i.e. the nurse-call systems with pressure mats) and there were limited data about the implementation of novel, emerging technologies. This likely reflects the embryonic stage of implementation of novel technologies within care homes. However, we found that the complex location-based system at Conifer Gardens had not been successfully implemented, at least in part because its myriad data collection capabilities were deemed not useful enough to persevere with workability challenges and financial cost. This suggests that complexity and cost may be substantially important factors in implementation that outweigh any potential benefits from enhanced clinical knowledge.

The most challenging aspect of the study was ascertaining directly a broader range of views of residents with cognitive impairments. This appears to reflect the increasingly high numbers of people with severe levels of cognitive impairment living in residential care. It also possibly indicates a scenario whereby monitoring technologies tend to be used in the care of residents with higher levels of impairment and complex co-morbidities.

Despite these limitations, this study benefits from multiple methods of data collection; in particular the iterative combination of observation and interview which afforded comprehensiveness and reflexivity. The use of Normalization Process Theory has highlighted the apparent importance to implementation of the involvement of stakeholders in discussions, decisions, and training which moves beyond functional instruction. Further research incorporating longitudinal tracking of an implementation process (i.e. from initial discussions within care homes prior to investing in a technology, through its introduction into practice, and subsequent evaluations of its impact) would be beneficial. Further research might also explore the relationship between specific diagnoses and stages of dementia and the implementation of monitoring technologies. Finally, exploration into the use of monitoring technologies in the observation and recording of staff activities would also appear relevant.

## 6 Conclusion

Monitoring technologies offer the potential to enhance safety and to enhance aspects of care such as freedom of movement for residents, or staff understanding of resident behaviours. However, implementation is likely to be influenced by the extent to which these technologies are perceived to enhance safety, with less consideration of wider benefits or of ethical challenges. Successful implementation may be facilitated by staff training that moves beyond functional instruction to include deeper discussion about anticipated benefits and challenges, and by the involvement of staff, residents and relatives in discussions and decision-making within the implementation process. The use of Normalization Process Theory has been particularly fruitful in highlighting the importance of the involvement of stakeholders as a facilitator of successful implementation.

## Acknowledgements

This work was supported by a Doctoral Training Grant from the UK Medical Research Council [grant number MR/K500823/1], and a University of Manchester President’s Award for Alex Hall. We would like to thank all the residents, relatives and staff for their willingness to participate in this study. We thank the owners and management of the three residential homes for their generous access to their facilities.

## Conflict of interest

None.

## Funding

UK Medical Research Council Doctoral Training Grant MR/K500823/1. No role in design or conduct of research.

## Ethical approval

NHS National Research Ethics Service Committee North West – Haydock (reference 13/NW/0752).
